# The effect of varying dietary nutrient densities on the relative growth of ostrich body components

**DOI:** 10.4102/jsava.v91i0.2029

**Published:** 2020-08-26

**Authors:** Tertius S. Brand, Werné J. Kritzinger, Leanne Jordaan, Louwrens C. Hoffman

**Affiliations:** 1 Department of Animal Sciences, University of Stellenbosch, Stellenbosch, South Africa; 2 Western Cape Department of Agriculture, Elsenburg, South Africa; 3 Centre for Nutrition and Food Sciences, Queensland Alliance for Agriculture and Food Innovation, University of Queensland, Coopers Plains, Australia

**Keywords:** >ratites, growth, nutrient densities, ostrich nutrition, poultry nutrition

## Abstract

The influence of varying dietary protein and energy levels on the relative growth of body components of ostriches was evaluated over a 244-day growth period. One hundred twenty 1-day-old ostrich chicks were randomly assigned to 15 pens. Three varying energy regimes (high, medium and low) and five protein levels (1–5) were supplied *ad libitum* to each pen. A randomly selected bird from each pen was slaughtered at 1, 35, 63, 103, 159, 168 and 244 days of age. Each bird was weighed, stunned, exsanguinated, defeathered and eviscerated. Individual body components were dissected and weighed at every slaughter age. Proximate analysis was performed on these components, which were ground with the remainder of the carcass, excluding gut content, but including blood and feathers. Based on the analysis of ostrich feathers and the known mass of the feathers, the protein mass contribution of the feathers was deducted from the protein accretion of the bird. All the data were transformed to natural logarithms and regressed against the featherless body protein growth. Intercepts and slopes were compared to determine differences in growth rate ascribed to nutrient densities. Neither dietary energy nor dietary protein level had a significant effect on the relative growth of the measured components in this study. Allometric coefficients were established, which could be helpful to improve the accuracy of simulation modelling attempts for ostrich nutrition.

## Introduction

The popularity of ostrich feathers in the fashion industry has led to the domestication of ostriches, and of late, their value has been increasing because of the importance being accorded to their skin and meat (Brand & Cloete 2015). Therefore, interest in commercial breeding and rearing of ostriches is growing in many countries worldwide (Cloete et al. 2012), and this increase in demand necessitates more research on these birds, especially with regard to their maintenance and nutritional requirements throughout various stages of their growth and production (Bovera et al. 2014; Brand, Olivier & Gous 2015). Nutrition is one variable that ostrich producers have most influence over, with energy and protein being the main nutrients in ostrich diets (Nikravesh-Masouleh et al. 2018). Knowledge of how the growth of body components is affected by varying the level of dietary energy and protein contents in diets will aid formulators with the optimisation of feed costs. An increase in body weight and whole body fat percentage is expected when a high-density diet is consumed (McDonald et al. 2002). This is not necessarily beneficial, as it points to the consumption of more than the required amount of nutrients at higher financial costs. Ostriches deposit fat reserves in the body cavity around the gut as a fat pad as well as subcutaneously (Brand et al. 2004; Cloete et al. 2006; Mellett 1992; Swart, Siebrits & Hayes 1993c). They also show great variability in gut fill (Cilliers et al. 1998; Swart, Mackie and Hayes 1993a), and this will influence the validity of the results found when working with live weight. Therefore, care must be taken while evaluating ostrich growth in terms of average daily gain because an increase in live weight and size does not necessarily signify an increase in commercially marketable products. Growth is influenced in various ways such as environmental conditions, stocking density and the welfare of the animal at certain stages of the growth cycle. Therefore, it is more accurate to work with weight units, as it improves the precision of measurement compared to measureing the size and length of the body parts of the live animal.

There is a need for a system to predict the nutritional requirements of ostriches in all the different phases of production. Such a system should be able to predict the effects of the environment, genotype and feed on the growth of the individual body components. This could be achieved by predicting body component growth as a proportion of the featherless empty body protein weight (EBPW) as measured in weight units (Danisman & Gous 2007). Individual component’s growth can be related to each other by relating these to a constant factor such as the EBPW because of the known relationships between the EBPW and the weighed components (Emmans 1989; Kritzinger 2011). Knowledge of the nutritional influence on the relationship of body components with EBPW will be valuable when modelling nutrient requirements, as it changes throughout the growth cycle. Accurate nutrient requirement standards can only be achieved when the growth of the body components and body protein from the feather protein are separate. This separation is necessary to enable simulation modelling which plays an important role in the future of ostrich production (Brand 2008).

Studies on the growth of ostriches in terms of carcass components and through all of the different growth stages and not just live weight and size measurements are scarce. Limited research is available on maximising growth in ostriches through dietary manipulation, and the effect of diet composition on the proportional change of chemical components in the whole body of the ostrich is unclear. Therefore, the aim of this study was to evaluate the effect of different dietary energy and proteins on the growth of carcass components as a proportion of the EBPW, and to determine if varying energy and protein levels could be used to manipulate component growth and the chemical composition of the body of the ostrich from pre-starter to finisher.

## Materials and methods

A total of 120 one-day-old ostrich chicks were placed in 15 pens, containing eight birds per pen. A 3 × 5 factorial design was used to allocate formulated diets with three energy regimes (high, medium and low) and five protein levels (1–5) (with accompanied amino acid inclusion levels) to each pen. Four stages (pre-starter, starter, grower and finisher) of feeding were used, and feed was supplied *ad libitum* throughout the trial (Table 1). The median feeding regime was chosen according to standard requirement levels for energy and protein (amino acids) (Cilliers et al. 1998; Smith et al. 1995). An average feed intake per bird (Table 2) was determined by weighing the supplied feed and the feed leftovers for each pen daily throughout each phase. The feeds were sampled and analysed (AOAC 2002) for protein, amino acids, fat, neutral detergent fibre (NDF), acid detergent fibre (ADF) and ash. Clean potable water was sufficiently available throughout the trial. A bird was selected at random from each of the 15 pens and slaughtered at 1, 35, 63, 103, 159, 168 and 244 days of age.

**TABLE 1:** Energy and protein contents (as is) of treatment diets used during the separate growth phases of ostriches.

Feeding regime	Energy level	Protein level	Growth phase							
			Pre-starter (0–1 month)		Starter (1–3 months)		Grower (3–6 months)		Finisher (6–9 months)	
			Energy content (MJ/kg feed)	Protein content (g/kg feed)	Energy content (MJ/kg feed)	Protein content (g/kg feed)	Energy content (MJ/kg feed)	Protein content (g/kg feed)	Energy content (MJ/kg feed)	Protein content (g/kg feed)
1	Low	1	12.5	170	10.5	140	8.5	115	7.5	80
2	Low	2	12.5	190	10.5	160	8.5	135	7.5	100
3	Low	3	12.5	210	10.5	180	8.5	155	7.5	120
4	Low	4	12.5	230	10.5	200	8.5	175	7.5	140
5	Low	5	12.5	250	10.5	220	8.5	195	7.5	160
6	Medium	1	14.5	170	12.5	140	10.5	115	9.5	80
7	Medium	2	14.5	190	12.5	160	10.5	135	9.5	100
8	Medium	3	14.5	210	12.5	180	10.5	155	9.5	120
9	Medium	4	14.5	230	12.5	200	10.5	175	9.5	140
10	Medium	5	14.5	250	12.5	220	10.5	195	9.5	160
11	High	1	16.5	170	14.5	140	12.5	115	11.5	80
12	High	2	16.5	190	14.5	160	12.5	135	11.5	100
13	High	3	16.5	210	14.5	180	12.5	155	11.5	120
14	High	4	16.5	230	14.5	200	12.5	175	11.5	140
15	High	5	16.5	250	14.5	220	12.5	195	11.5	160

**TABLE 2:** The average feed intake of the birds during the pre-starter, starter, grower and finisher phases at various energy and protein levels in the formulated diets.

Feeding regime	Energy level	Protein level	Feed intake (g/bird/day)			
			Pre-starter (0–1 month)	Starter (1–3 months)	Grower (3–6 months)	Finisher (6–9 months)
1	Low	1	392	556	2526	4778
2	Low	2	421	907	2581	4356
3	Low	3	445	796	2577	4217
4	Low	4	353	873	2530	4001
5	Low	5	384	723	2959	4038
6	Medium	1	433	539	2210	3854
7	Medium	2	489	612	2162	3447
8	Medium	3	209	742	2264	2997
9	Medium	4	473	486	2249	3808
10	Medium	5	228	531	2690	3602
11	High	1	195	581	2292	3210
12	High	2	212	486	2131	2909
13	High	3	162	438	2191	2992
14	High	4	133	891	2004	2815
15	High	5	161	464	1710	2773

At each slaughter age, birds were weighed, stunned, exsanguinated, defeathered and eviscerated according to standard South African ostrich slaughter techniques, as described by Hoffman (2012). During exsanguination, the blood from each ostrich was collected in a separate container. Following exsanguination, the feathers were plucked and skins were carefully flayed from the carcass. The intestines, heart, liver and 13 individual muscles (Table 3) were removed and weighed. The same procedure was carried out for the femur, rib cage, neck, tibiotarsus and wingtip bones that were collected. After weighing, all the individual components along with the blood and the feathers were frozen in separate plastic bags for each bird until grounding commenced. All the body parts were ground and mixed thoroughly after which randomly selected samples (approximate 150 g) were used to perform a proximate analysis (AOAC methods, 2002), yielding the chemical composition of the entire bird.

**TABLE 3:** Allometric coefficients relating the natural logarithm of muscle weight to the natural logarithm of empty body protein weight.

Muscle (commercial name)	Constant term	Regression coefficient	*R*^2^
*M. iliotibialis cranialis* (top loin)	‒3.2889 ± 0.0371	1.0834 ± 0.0212	0.96
*M. iliofemoralis externus* (oyster)	‒4.3223 ± 0.0312	1.2045 ± 0.0178	0.98
*M. iliotibialis lateralis* (round; rump steak)	‒2.5638 ± 0.0405	1.1276 ± 0.0232	0.96
*M. iliofibularis* (fan fillet)	‒2.37611 ± 0.0245	1.1607 ± 0.0140	0.99
*M. iliofemoralis* (eye fillet; inside strip)	‒3.7584 ± 0.0332	1.1884 ± 0.0190	0.97
*M. flexor cruris lateralis* (triangle steak; outside strip)	‒3.6823 ± 0.0529	1.1164 ± 0.0302	0.93
*M. obturatorius medialis* (long fillet; tenderloin)	‒3.9692 ± 0.0536	1.3710 ± 0.0306	0.95
*M. femorotibialis medius* (moon steak; tip trimmed)	‒4.4431 ± 0.0443	1.1302 ± 0.0254	0.95
*M. femorotibialis accessories* (tip)	‒2.5680 ± 0.0264	1.0253 ± 0.0151	0.98
*M. gastrocnemius pars interna* (big drum; inside leg)	‒2.5991 ± 0.0315	1.1027 ± 0.0180	0.97
*M. gastrocnemius pars media* (steak)	‒3.2352 ± 0.0347	1.1780 ± 0.0198	0.97
*M. gastrocnemius pars externa* (flat drum; outside leg)	‒2.9485 ± 0.0358	1.0762 ± 0.0205	0.96
*M. fibularis longus* (drum steak; mid leg)	‒3.4922 ± 0.0347	1.0277 ± 0.0199	0.96

*R*^2^, Coefficient of determination.

As growth is non-linear (Huxley 1932; Lawrie 1998; McDonald et al. 2002), all the data were transformed into the natural logarithmic form to obtain linear data. Swart et al. (1993a) observed considerable quantities of gut fill (8% – 15% of live weight), and also noted that major variation can occur in gut fill between individuals at any specific time. This motivated the use of empty gut weight rather than live weight. The feathers were analysed together with the remainder of the body in the current study. The proportional protein contribution of the feathers calculated according to Brand (2008) was deducted from the protein analysis obtained, as Emmans (1989) reported that feather protein and body protein should be evaluated separately. This yielded an estimation of the featherless EBPW that was used for further statistical analyses. Using the mixed models procedure of SAS statistical software version 9.1 (SAS 2000), the data were analysed to check for treatment differences by comparing the slopes and the intercepts independently. Significance level was declared at *p* ≤ 0.05. Treatments were taken as the combinations of different levels of dietary energy (low, medium or high) and protein (level 1–5). Where no differences were found between treatments, a general regression line was fitted to the data. This method of analysis was repeated for all the measured components.

### Ethical consideration

Ethical approval to conduct the study was obtained from the Departmental Ethical Committee for Research on Animals (DECRA), Western Cape Department of Agriculture (project no. R10/13).

## Results

Differing dietary protein levels were found to have no effect on body weight or any of the body components in this study and were thus excluded from any further analyses.

The effects of the different dietary energy regimes on the weighed muscles, bones and organs are given in Tables 3–5, respectively. No differences were found between any of the muscles for the different dietary energy levels. For each muscle, the treatment groups were therefore pooled to give a linear regression to describe the change in weight for each muscle measured (Table 3).

**FIGURE 1:**
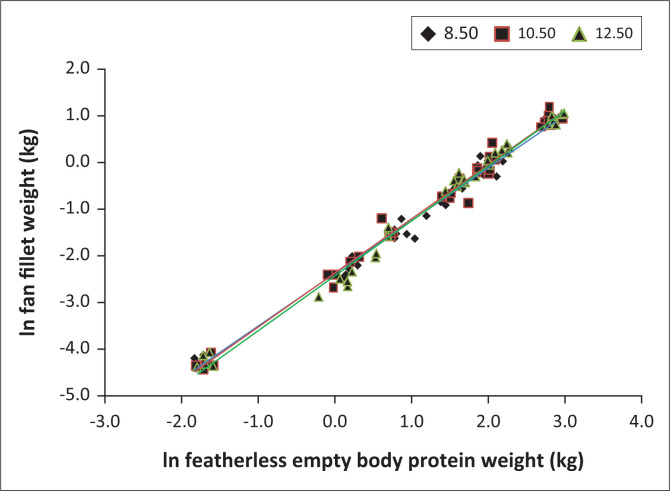
Regression lines fitted to the natural logarithms of the *M. iliofibularis* (fan fillet) weights (kg) against the natural logarithms of the empty body protein weight (kg) for the different dietary energy levels (8.5, 10.5 and 12.5 MJ ME/kg).

Figure 1 illustrates the fitted and compared regression lines for the *M. iliofibularis* (fan fillet). The lack of differences in the slopes and/or intercepts is apparent. In the cases where no differences were found for the individual muscles, a common regression line was fitted to the data to simplify future applications of the results.

The allometric relationships between the weighed bones (femur, rib cage, patella, neck, tibiotarsus and wingtip) and EBPW are given in Table 4.

**TABLE 4:** Allometric coefficients relating the natural logarithms of the weighed bone components to the natural logarithms of empty body protein weight with dietary energy levels as groups to determine the effects on component growth (kg).

Bones	Constant term	Regression coefficient	*R*^2^
Femur	‒1.6565 ± 0.0441	1.1030 ± 0.0252	0.95
Rib cage	‒0.2894 ± 0.0429	1.0782 ± 0.0242	0.95
Patella	‒3.8800 ± 0.0373	1.1741 ± 0.0213	0.97
Neck	‒2.0512 ± 0.0226	1.0013 ± 0.0129	0.98
Tibiotarsus	‒2.0010 ± 0.0903	1.1423 ± 0.0516	0.83
Wingtip	‒3.8174 ± 0.0423	1.2407 ± 0.0242	0.96

*R*^2^, Coefficient of determination.

The allometric coefficients between some of the ostrich organs and EBPW are shown in Table 5. Increasing the dietary energy levels had no effect on the relationship between organ weights and EBPW. Although the different levels of dietary energy did not alter the allometric relationships (of liver weight to EBPW), a proximate analysis on the liver was not performed in this investigation.

**TABLE 5:** Allometric coefficients relating the natural logarithm of the organ weights (kg) to the natural logarithm of empty body protein weight (kg) with dietary energy levels as groups to determine the effect on component growth (kg).

Some organs	Constant term	Regression coefficient	*R*^2^
Heart	‒3.0500 ± 0.0358	1.0977 ± 0.0205	0.97
Intestines	‒1.4941 ± 0.0479	0.9034 ± 0.0479	0.92
Liver	‒1.9477 ± 0.0314	0.9652 ± 0.0180	0.96
Stomach	‒2.7089 ± 0.0502	1.0335 ± 0.0287	0.93

*R*^2^, Coefficient of determination.

The differences between the chemical components (as is) of the whole body as a result of increasing dietary energy levels are shown in Table 6. Increases in dietary energy did not affect moisture or ash content when regressed against EBPW. Fat deposition was altered and increased as the energy level in the diet increased.

**TABLE 6:** Allometric coefficients relating the natural logarithms of the chemical component growth (kg) in the whole body (as is) to the natural logarithms of empty body protein weight (kg) with dietary energy levels as groups.

Body composition	Energy level	Moisture	Fat	Ash
Constant term	Low	-	‒1.6434† ± 0.1955	-
	Medium	1.4013 ± 0.0168	‒1.4322‡ ± 0.1956	‒1.5312 ± 0.0257
	High	-	‒1.0468§ ± 0.1380	-
Regression coefficient	Low	-	0.8301† ± 0.1058	-
	Medium	0.8685 ± 0.0091	1.0129†† ± 0.1069	1.0970 ± 0.0141
	High	-	1.1531‡ ± 0.0756	-
*R*^2^	-	0.99	0.84	0.98

†, ‡, § , Values with the same superscript in the same column (per coefficient) do not differ significantly (*p* ≤ 0.05).

## Discussion

The objective of this study was to establish whether the relationships between the physical body components and the EBPW were affected by differing dietary energy and protein levels. Differences in the relationships, as a result of nutrition, would therefore imply that tissues other than protein (such as lipid) are deposited in or with the component to increase the weight thereof.

The results of this study with regard to protein content of diets confirm the findings of others (Brand et al. 2003; Brand, Nel & Van Schalkwyk 2000; Gandini, Burroughs & Ebedes 1986; Nikravesh-Masouleh et al. 2018; Swart & Kemm 1985) that different levels of protein within certain ranges and above minimum levels for the growth phase in ostrich diets did not have a significant effect on the growth rate (live weight increase over time) or body measurements. Mahrose et al. (2015) and Abd El-Hack and Amer (2019) concluded that young ostrich chicks (pre-starter) can grow on relatively low crude protein content diets of 18%, which is between the lowest levels, 1 and 2, of protein content in this study.

Various reports noted increased growth rates for ostriches fed higher energy diets compared to a lower energy level: Swart and Kemm (1985) fed energy levels of 8.1, 9.5 and 10.7 MJ ME/kg feed to slaughter birds; Cornetto, Angel and Estevez (2003) supplied ostriches up to 148 days of age with dietary energy of 11.71, 12.90 and 14.09 MJ ME/kg feed; Brand et al. (2000) provided ostrich chicks (13–34 kg in mass) with energy levels of 10.5, 12.5 and 14.5 MJ ME/kg feed; Brand et al. (2003) fed diets varying in energy levels, 8.5, 10.5 and 12.5 MJ ME/kg feed. It is worth noting that in contrast, Tasirnafas et al. (2015) evaluated the effect of different levels of dietary vegetable wastage and energy on ostrich chicks and found that energy level (10.5 and 11.3 MJ/kg) had no effect on feed intake, weight gain or feed efficiency. More recently, Nikravesh-Masouleh et al. (2018) found that increasing energy and protein levels led to decreased weight gain in ostrich chicks (2–9 weeks of age). They found that the most important parameters at 9 weeks of age, such as breast, abdomen and thigh circumference as well as body length, were higher in birds that were fed the lower energy content diet (10.1 MJ/kg) as supposed to a higher energy diet (10.9 MJ/kg), although greater tail length and shank circumference were observed in birds that were fed the higher energy diet.

The apparent increase in growth rate that accompanies increasing dietary energy levels in repeated measurement trials, as reported by Swart and Kemm (1985), Cornetto et al. (2003) and Brand et al. (2000, 2003), can partly be ascribed to an increase in fat deposition in the body and the subsequent changes in body composition as a whole. Ostriches are reported to deposit vast amounts of fat reserves subcutaneously and in the body cavity around the gut (Brand et al. 2004; Cloete et al. 2006; Swart et al. 1993c). Although body fat is expected to increase relatively as the bird matures (Degen et al. 1991; Huxley 1932; Lawrie 1998; McDonald et al. 2002), the presence of increasing levels of dietary energy contributed to significant increases in fat deposition in this study. The effect of fat deposition in growth studies where animals consume more nutrients than they require for maintenance should not be overlooked. As the organs (heart, intestines and stomach) are expected to consist mostly of protein, the results are as expected. The protein content of the individual muscles in this study was not determined, and the variable amounts of lipid deposited in the muscles because of normal growth and treatment differences are thus not accounted for, but differences because of diet could be expected to be minimal as Hoffman and Fisher (2001) and Sales and Hayes (1996) reported small amounts of fat deposition in ostrich muscle.

The energy cost of body protein accretion is greater than that required for fat deposition (Swart et al. 1993c). The efficiency of metabolisable energy utilisation in the ostrich is a complex concept as the ratio of energy directed towards the separate deposition of protein and fat changes in maturing animals (Swart et al. 1993c). Decreasing dietary energy levels frequently amounts to increasing levels of crude fibre in diet formulations. This increases the passage rate of the digesta, and more nutrients will pass through the small intestine (Just 1982; Just, Fernandez & Jorgensen 1983). An increase in intestinal passage rate will shorten the time that the digesta are subjected to microbial hindgut fermentation (Swart, Siebrits & Hayes 1993d).

Swart, Mackie and Hayes (1993b) reported that fermentative digestion of fibre (cellulose and hemicellulose) can contribute to the energy requirements of the growing ostrich in the form of volatile fatty acids (VFAs). Musara et al. (2003) defined the mechanism for VFA uptake from the hindgut as H^+^-K^+^-ATPase activity that utilises H^+^-ions from a source other than the hydration of CO_2_. The uptake of VFA from the ostrich hindgut is thus a process of secondary active transport, which is highly dependent on intracellular hydrogen ion generation (Musara et al. 2003). The manipulation and improvement of VFA uptake could lead to improved energy efficiency with economic advantages.

The question raised by Swart et al. (1993a) regarding the efficiency of the utilisation of energy absorbed from the foregut and the hindgut remains unanswered. The protein:fat deposition rate, as determined by factors such as genotype, environmental conditions and stress susceptibility, changes in the maturing animal. This will cause changes in the way that absorbed energy and non-limiting protein are directed towards maintenance and growth functions. Defining the mechanisms and factors affecting the relative protein:fat deposition rate and the efficiency of energy uptake and utilisation is vital for the accurate determination of the changing nutrient requirements of the ostrich.

No changes to the relationships of the individual bone components and EBPW were anticipated in this study, as bone density is expected to remain constant. Almeida Paz et al. (2008) suggested that live weight was the only factor that influenced the bone quality traits in their study.

Perhaps, other nutrients and not just energy and protein should be evaluated in future studies; for example, Kaimi-Kivi et al. (2015) found that the addition of probiotics to the feed of pre-starter ostrich chicks could modulate specific body characteristics by increasing the trunk volume, which might indicate higher meat deposition and higher total body area which, in turn, might indicate more skin from chicks fed with probiotics.

## Conclusion

The effect of varying diets on some muscles, bones and organs of the ostrich was investigated. It was shown that increasing dietary energy and protein levels did not affect the weight of the individual body components when expressed as allometric equations of EBPW. Even though no differences were found between treatments in this study, the general regression line that was fitted to the data provides simulation modellers with an equation to compare and predict carcass component growth by utilising the existing allometric relationships between component and EBPW growth. This would be helpful in ostrich nutrition and production. Protein deposition is the main factor that determines live weight gain, whilst the energy cost of protein deposition is greater than that required for fat deposition. Consequently, the effect of higher energy intakes on the protein deposition rate in the body is not yet known, as dietary protein and amino acid levels, as used in this study, had no effect on the weighed variables. More research on the exact combining effects of dietary energy and protein, the efficiency of nutrient utilisation and the possible manipulation of these factors is required.
